# The effect of intervention using an emotional recognition coaching companion robot on the elderly people with depression

**DOI:** 10.1192/j.eurpsy.2023.252

**Published:** 2023-07-19

**Authors:** K. Kim, B.-H. Yoon, Y.-H. Sea, J.-H. Song

**Affiliations:** Naju National Hospital, Naju, Korea, Republic Of

## Abstract

**Introduction:**

During the COVID-19 pandemic, care for the elderly in the community was greatly limited. Accordingly, the demand for alternative community care have increased to cope with changing situations.

**Objectives:**

In this study, we tried to find out whether the companion robot improved mood state and related problem in depressive or isolated community dwelling elderly

**Methods:**

For 186 community dwelling elderly who have received social welfare service due to depression or social isolation, we provided companion robot that could support their daily living. The robot was equipped with special program that could recognize and respond to the participant’s own emotion. It was part of behavioral activation techniques which is one of powerful treatment for depression. The self-report questionnaires were used to measure changes in cognitive function, depression, suicidality, loneliness, resilience and satisfaction of life. Outcomes were measured before using companion robot and after 3 months, and we compared them.

**Results:**

The elderly using companion robot for 3 months showed improved cognitive function (45.7% to 30.1%), depression (p<0.001), suicidality(p<0.001), and loneliness (p=0.033) in the self-report questionnaire. Resilience(p=0.749) and satisfaction of life (p=0.246) were also improved but not reached significance.

**Image:**

**Figure:**
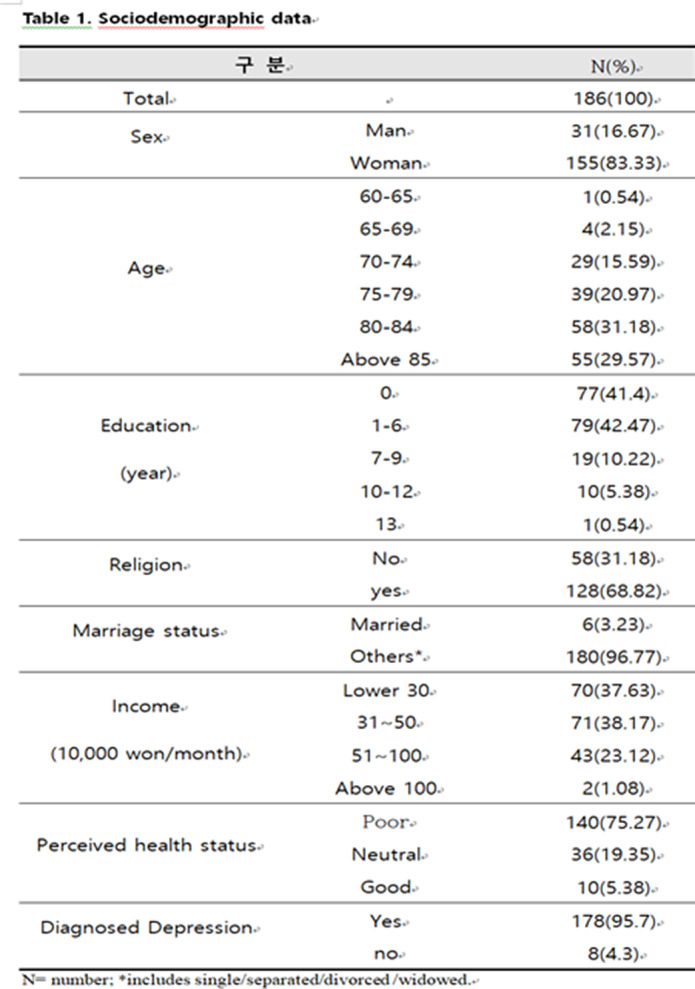

**Image 2:**

**Figure:**
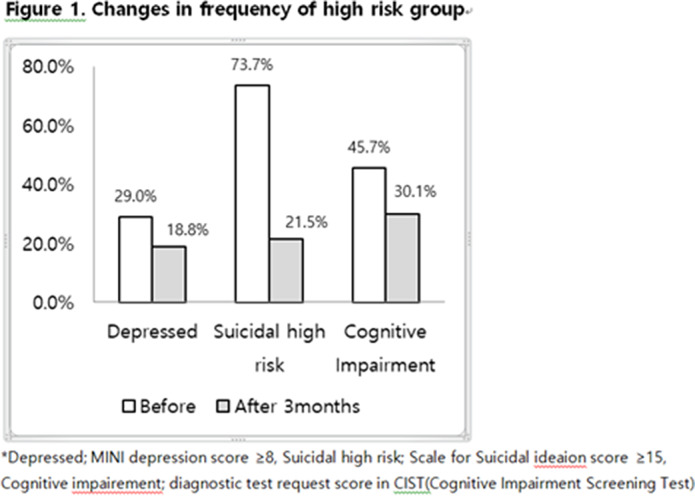

**Image 3:**

**Figure:**
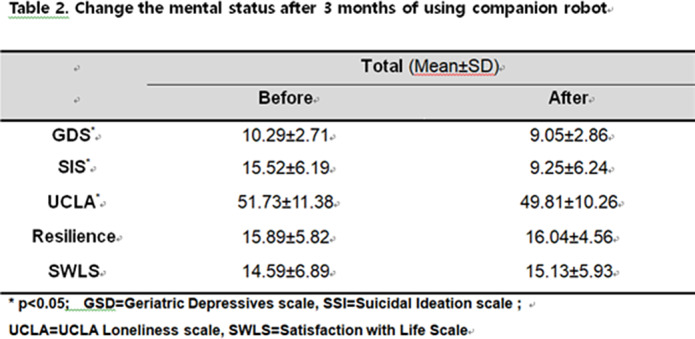

**Conclusions:**

These findings showed that the use of companion robot with emotional recognition coaching program could help improve depression, cognitive function, loneliness and suicidal ideation. In particular, this effect was also useful for those who were diagnosed with depression. Also if we can put more techniques of behavioral activation programs into robot, it could be useful in community care for depressive and isolated elderly.

**Disclosure of Interest:**

None Declared

